# Potassium Permanganate in Amenorrhœa

**Published:** 1886-09

**Authors:** Benjamin Marshall

**Affiliations:** Col. Phys. and Surg., N. Y.; 924 Suter Street, San Francisco, Cal.


### *POTASSIUM PERMANGANATE IN AMENORRHŒA.*

*To the Editors of the Therapeutic Gazette:*

GENTLEMEN:—In your June issue is an article on the use of permang. pot. as an emmenagogue, and asking for results. My conclusions, after using it in about fifty cases, are as follows:

1. It acts with certainty in about seventy per cent. in selected cases.

2. It may be given at any time, but preferably one or two hours after eating, as it is not so apt to sicken the stomach. I don't think it is any more efficient when given on an empty stomach.

3. I have used the sulphate in a number of cases with less certain effect; the result attained, however, may have been accidental.

4. In most cases it produces an exhilaration of spirits.

5. It has a decided tonic effect.

6. It will become an indispensable therapeutic agent.

7. Its disagreeable effect on the stomach is best relieved by a combination of

| | | |
|---|---|---|
| Cerri oxalat., - - - - | gr. i. | |
| Cocaine hyd. chlor., - - - - | gr. 1/6 | |
| Bismuth, subnit., - - - - | gr. v. | |
| Pulv. ipecac, - - - - - | gr. 1/10 | M. |

Ft. cachets, one every two hours.

Respectfully,

BENJAMIN MARSHALL, M. D.,

*Col. Phys. and Surg., N. Y.*

924 Suter Street, San Francisco, Cal.

—*Therapeutic Gazette.*

